# Hospitalization and Death Acceptance: A Meta‐Ethnography of Perceptions of Hospital as a Place of Death for Nursing Home Residents

**DOI:** 10.1111/opn.70101

**Published:** 2026-10-04

**Authors:** Bridget Laging, Rachel MacLean, Jessica Dickson, Janine Alan

**Affiliations:** ^1^ Alfred Emergency Academic Centre Alfred Health Melbourne Victoria Australia; ^2^ School of Public Health and Preventive Medicine Monash University Melbourne Victoria Australia; ^3^ Department of Interdisciplinary Studies, 3 Bailey Drive University of New Brunswick Fredericton New Brunswick Canada; ^4^ RMIT University Library Melbourne Victoria Australia; ^5^ School of Population and Global Health The University of Western Australia Perth Western Australia Australia

**Keywords:** aged care, dying, emergency department, end‐of‐life, systematic review, terminal

## Abstract

**Background:**

Efforts to reduce hospital transfers from nursing homes at the end of life have received considerable attention. However, less is known about how hospitals are understood and experienced as places of death for nursing home residents.

**Objective:**

To synthesize qualitative evidence exploring stakeholder perceptions of hospital as a place of death for older adults living in nursing homes.

**Design:**

A meta‐ethnography of qualitative evidence following the Meta‐ethnography Reporting Guidelines (eMERGe). Four bibliographic databases (MEDLINE, CINAHL, PsychINFO and Scopus) were systematically searched to identify all studies published from January 2012 to March 2024.

**Review Methods:**

Studies were included if they reported qualitative findings on nursing home residents dying in hospital. Following quality appraisal, first‐ and second‐order constructs were extracted and translated across studies using reciprocal and refutational analysis to develop third‐order interpretations.

**Findings:**

Two overarching interpretive themes were generated: (1) *Controlling Symptoms of Dying: Comfort and Discomfort*; and (2) *Acceptance of Death After All Efforts to Save Life Have Been Exhausted*. Hospitals were perceived as both impersonal environments and medically resource‐rich settings that legitimize death through medical authority.

**Conclusions:**

Hospitals occupy a paradoxical position in end‐of‐life care for nursing home residents. While often viewed as clinically oriented and less personal, they are also perceived to provide symptom control and reassurance that ‘everything possible’ has been done. Addressing these perceptions—alongside strengthening palliative capacity within nursing homes—is essential to supporting residents' preferences and enabling high‐quality end‐of‐life care across settings.

**Implications for Practice:**

Nurses can support more person‐centred end‐of‐life care by strengthening palliative symptom management in nursing homes, engaging residents and families in early advance care planning, and improving communication about dying and hospital treatment. These practices may help reduce avoidable hospital transfers and support residents to die in their preferred place.

## Summary

**What does this research add to existing knowledge in gerontology?:**

- Moves beyond measuring hospitalization rates by exploring how residents, families and clinicians perceive hospitals as places of death, providing a deeper conceptual understanding of end‐of‐life transitions from nursing homes.
- Identifies a paradoxical view of hospitals: they are seen as resource‐rich settings capable of relieving suffering but also impersonal, clinical environments that may reduce comfort and dignity.
- Shows hospital transfer often has a symbolic role, legitimizing death by demonstrating that all possible treatments were attempted, reflecting broader societal medicalization of dying.

**What are the implications of this new knowledge for nursing care for and with older adults?:**

- Nursing homes must strengthen palliative care capacity and symptom management, including staffing, training and medication access, to address perceptions that hospitals provide better comfort at end of life.
- Nurses should engage residents and families in earlier, holistic advance care planning, including education about the dying process and realistic expectations of hospital treatments.
- Improved recognition of the dying phase and communication with families can help prevent unnecessary hospital transfers and support residents to die in their preferred place.

**How could the findings be used to influence practice, education, research, and policy?:**

- Policies should recognize dying as an expected outcome in nursing homes and ensure consistent access to palliative care resources, specialist support and adequate staffing to reduce avoidable hospital deaths.
- Practice changes should focus on integrated care between hospitals and nursing homes, stronger symptom management protocols and clearer communication about goals of care during clinical deterioration.
- Future research and education should explore hospital clinicians' perspectives, symptom triggers for transfers and training in end‐of‐life communication and care for professionals working with older adults.

## 1 Introduction

The transition between nursing homes (such as long‐term care facilities, residential aged care facilities and care homes with 24‐h nursing staff) and hospitals at the end of life has received increasing international attention from policymakers, health service providers and researchers. Many older people living in nursing homes (hereafter referred to as residents) are transferred to a hospital in their final weeks or days. To date, efforts have largely focused on reducing hospital transfers at the end of life. Far less attention has been given to how hospitals are perceived as a place of death for nursing home residents. Understanding these perceptions is essential for shaping end‐of‐life care that aligns with residents' needs, preferences and rights.

### 1.1 Residents Dying in Hospitals

Hospitalizations and in‐hospital deaths among residents remain common, though rates vary widely across settings and studies. A systematic review reported that between 25% and 70% of residents were hospitalized in the last month of life, and between 6% and 77% died in hospital—often shortly after admission (Allers et al. 2019). Similar variation is confirmed in other systematic reviews (Costa et al. 2016; Hoffmann and Allers 2020; Lemoyne et al. 2019).

Both individual and system‐level factors shape these outcomes. Younger age, male sex, dementia, stroke, cancer and longer nursing home stays are associated with in‐hospital death (Costa et al. 2016; Hoffmann and Allers 2020), although the presenting symptoms precipitating hospital transfers remain underreported (Allers et al. 2019; Costa et al. 2016; Hoffmann and Allers 2020; Lemoyne et al. 2019). At the system level, advance care planning is consistently associated with fewer in‐hospital deaths (Hoffmann and Allers 2020; Searle et al. 2023; Costa et al. 2016), whereas limited access to registered nurses, visiting doctors and medical resources increases hospital transfers (Laging et al. 2015, 2018). These findings suggest that hospital death is not inevitable but rather produced through modifiable organizational and cultural practices.

Yet, while we know much about *rates* and *risk factors*, we know comparatively little about how hospital transfers and hospital deaths are experienced and understood by those most affected.

### 1.2 Palliative Care and the Medicalization of Dying

Nursing homes are well positioned to provide end‐of‐life care, given the high prevalence of frailty and end‐stage disease among this population (Cardona‐Morrell et al. 2017). However, research suggests that palliative approaches, defined by the World Health Organization as an approach to improving individuals facing life‐threatening problems' quality of life (World Health Organization 2020), are often initiated only as end‐of‐life care (Cable‐Williams and Wilson 2017), or care provided during the last days, weeks or months of life when curative treatment is no longer possible (Huffman and Harmer 2024).

Although palliative care emphasizes quality of life and symptom relief alongside treatment (World Health Organization 2020), many healthcare systems continue to frame death as something to be resisted and postponed (Clark 2002). This framing contributes to the medicalization of dying and the persistence of hospital‐based interventions at the end of life (Lofland 2019; Sallnow et al. 2022), and to the difficulty for providers, residents and families alike in accepting the natural process of death (Clark 2002; Sallnow et al. 2022). As a result, hospitals occupy an ambiguous position: they may represent technological rescue and clinical expertise but also invasive intervention, disruption and dislocation from familiar surroundings.

### 1.3 The Unresolved Question: Is Hospital a ‘Good’ Place to Die?

Conditions associated with a ‘good death’ include dying in one's preferred place, relief from pain and other symptoms, avoidance of unnecessary life prolongation, effective communication, emotional support and opportunities for cultural, religious or spiritual practices (Zaman et al. 2021). While these principles are increasingly emphasized, little is known about how hospitals are perceived in relation to these conditions—whether as settings that hinder or support good deaths for nursing home residents. Addressing this gap is essential, as hospitals may simultaneously be seen as overmedicalized sites of dying and as places with access to expertise and resources. Despite this complexity, no synthesis has systematically examined how hospitals are perceived as places of death for nursing home residents.

### 1.4 Study Aim and Contribution

This meta‐ethnography synthesizes qualitative evidence on how hospitals are perceived as places of death for nursing home residents, drawing on the perspectives of residents, families and healthcare providers. By interpreting and translating findings across studies, this synthesis seeks not merely to aggregate experiences but to generate a deeper conceptual understanding of the meanings attached to hospital death.

In doing so, this study moves beyond the policy question of how to reduce transfers and instead interrogates the underlying assumptions about where and how older people ‘should’ die. The findings will inform more nuanced, person‐centred and contextually responsive models of end‐of‐life care across institutional boundaries.

## 2 Methods

### 2.1 Design

This qualitative meta‐synthesis employed meta‐ethnography as an inductive, interpretive analytical approach to generate deeper understanding of existing evidence on perceptions of hospital as a place of death for older people living in nursing homes (Noblit and Hare 1999). In meta‐ethnography, findings from primary studies are reinterpreted across three levels: *first‐order constructs* (participant quotations), *second‐order constructs* (themes and concepts developed by the original researchers) and *third‐order interpretations* (new, higher‐order explanations developed through synthesis) (Noblit and Hare 1999). By moving beyond aggregation, meta‐ethnography enables the development of new insights that have greater explanatory power than individual studies alone. This has been described as producing a new ‘storyline’ (Noblit and Hare 1999) or overarching explanation of a phenomenon (France et al. 2019). The review was conducted and reported in accordance with the eMERGe reporting guideline for meta‐ethnography (France et al. 2019) (see Supporting Information, Table S1).

### 2.2 Ethical Considerations

Formal ethics approval was not required for this study, as it is a systematic review and meta‐ethnography of previously published research. No new data were collected directly from human participants, and all included studies had already obtained ethics approval at the time of their original conduct. Nevertheless, given the sensitivity of the topic—perceptions of nursing home residents' deaths in hospitals—we approached the review with particular care. Our analytic and reporting processes were guided by a commitment to respectful representation of participants' experiences and by sensitivity to the emotional, cultural and ethical dimensions of death and dying.

### 2.3 Search Strategy

We searched four bibliographic databases (MEDLINE (Ebsco), CINAHL Complete (Ebsco), APA PsycINFO (Ebsco) & Scopus), chosen for their size and topic appropriateness. We conducted systematic searches of keywords and indexed terms with the assistance of a health librarian (JD). The search process was clustered into three concepts using the PICo (Population, Interest, Context) framework: (1) nursing home residents; (2) end‐of‐life death in hospital; (3) nursing homes and acute care hospital settings. A broad range of keywords using phrase searching and truncation were searched in MEDLINE in title OR abstract fields to ensure specific results and were combined with appropriate subject headings. The search was modified according to the indexing systems of the three other databases and was conducted between January 2023 and March 2024. Our search results were limited from January 2012 to March 2024 to synthesize research conducted within contemporary palliative‐integrated long‐term care contexts. The search strategy for each database is reported in Table S2. Lastly, we searched the reference lists and full texts of selected papers to identify additional relevant records that were missed by the electronic database search.

### 2.4 Inclusion/Exclusion

Studies were included that used qualitative methods through virtual or face‐to‐face contact and semi‐structured or open‐ended questioning approaches to explore perceptions of nursing home residents dying in the hospital. All qualitative data from qualitative descriptive or mixed methods studies were included. All studies were published in English in peer‐reviewed journals. We excluded case reports, reviews or systematic literature reviews, opinion pieces, editorials, comments, news, letters, conference abstracts and quantitative studies. We included older people living in nursing homes (permanent and respite) and excluded any community‐dwelling older people, people living in retirement villages or independent living units. Studies were only included if they contained perceptions of hospitals as a place of nursing home resident deaths (i.e., participant quote or researcher interpretation of participant quotes), including but not limited to perceptions from health care providers and consumers.

### 2.5 Study Selection

All identified records were initially imported into EndNote 20 bibliographic software (Clarivate Analytics) and then uploaded into Covidence systematic review software (Ventas Health Innovation, Melbourne, Australia) to streamline the systematic screening process. After removing duplicates, all titles and abstracts were screened by three reviewers (BL, JA, JD). Full texts of articles were screened by two independent reviewers (BL and RM) against the inclusion criteria. All qualitative studies that met the inclusion criteria were included. Any disagreements throughout the selection process were resolved within the team through consensus.

### 2.6 Quality Appraisal

To evaluate the extent to which each study reflected the key principles that underpin qualitative research (including rigour, trustworthiness and reflexivity), we utilized the Critical Appraisal Skills Programme for qualitative studies (CASP) (Critical Appraisal Skills Programme 2023). Two team members (BL and RM) independently assessed individual study quality and resolved divergent results together. We did not exclude any studies based on study quality.

### 2.7 Data Extraction

The full texts of all included manuscripts were read by two reviewers (BL and RM). Data on study characteristics (aims, methodology, sampling, data collection, analysis, setting and sample) were extracted by RM and cross‐checked by BL. All data about nursing home residents dying in a hospital were extracted into a data extraction tool in the latest version of Microsoft Word. We developed our data collection tool according to guidelines for literature synthesis for health research meta‐ethnographies outlined by Sattar et al. (2021). To preserve the context and meaning of the concepts, data directly relating to nursing home residents dying in hospitals were extracted into two columns: first‐order data (participant quotes) and second‐order data (categories, themes and/or original authors' interpretations).

### 2.8 Data Synthesis and Interpretation

Initial coding was undertaken alongside data extraction by creating two additional columns in the extraction table, where each reviewer (BL and RM) independently recorded initial codes and interpretations in relation to the first‐ and second‐order data. Using a constant comparative approach, we translated emerging third‐order concepts across studies to move beyond the original findings. This process involved examining whether study data could be understood as reciprocal translations (directly comparable) or refutational translations (oppositional). Noblit and Hare (1999) describe this as a ‘key judgment call’ (p. 81), requiring reviewers to determine relationships between studies in order to synthesize them. Identifying both reciprocal and refutational findings enabled the development of third‐order concepts that captured shared meanings as well as divergent and, at times, conflicting, perceptions of nursing home residents dying in hospitals.

To enhance trustworthiness, researchers engaged in an independent and reflexive analytic process, moving iteratively between emerging interpretations and the primary data, including coded findings. This allowed for careful examination and refinement of themes while acknowledging the influence of the reviewers' perspectives. Following a constructivist lens, we recognize that alternative research teams may have generated different interpretations (Noblit and Hare 1999). The first and second authors brought complementary clinical and research experience in both nursing home and hospital settings, offering a dual perspective that enriched analysis. Neither author was employed by a healthcare organization at the time of the review. Throughout the process, the author team met regularly to refine third‐order concepts, critically examining assumptions, biases and values that could shape the synthesis.

## 3 Results

### 3.1 Study Characteristics

A total of 12,473 studies were identified from the database searches, outlined by the Preferred Reporting Items for Systematic Reviews and Meta‐Analyses diagram in Figure 1 (Page et al. 2021). After de‐duplication, 6406 abstracts were screened, 720 full texts were reviewed, and 10 qualitative studies met inclusion criteria for this review. No additional sources were identified from the reference lists of included studies. The included studies were conducted in the USA (Akunor et al. 2022; Sopcheck and Tappen 2023; Stephens et al. 2015), France (Economos et al. 2020; Kouyoumdjian et al. 2019), England (Murphy‐Jones and Timmons 2016), Scotland (Alcorn et al. 2020), Australia (Lane and Philip 2015), the Czech Republic (Pechová and Loučka 2023) and Belgium (Reyniers et al. 2014).

**FIGURE 1:**
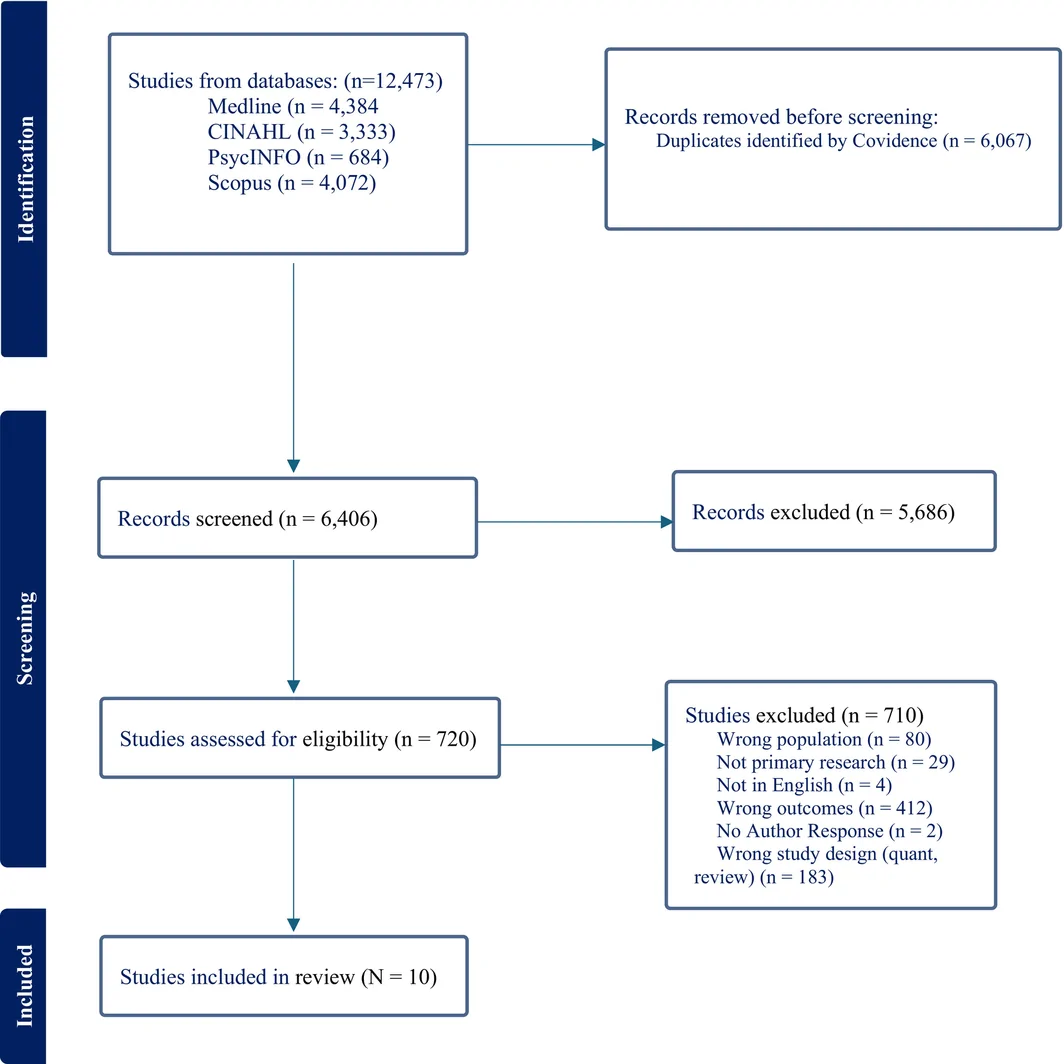
PRISMA flow diagram.

Detailed descriptions of the characteristics of the included studies are provided in Table 1. Most of the studies utilized purposive sampling and semi‐structured interviews. In our initial search, we were interested in any and all perceptions of hospital as a place of death for people from a nursing home. However, all studies identified in our search were focused on health care providers and consumers' perceptions. Nine studies focused on participants from nursing homes (Akunor et al. 2022; Alcorn et al. 2020; Economos et al. 2020; Kouyoumdjian et al. 2019; Lane and Philip 2015; Pechová and Loučka 2023; Reyniers et al. 2014; Sopcheck and Tappen 2023; Stephens et al. 2015) and one study focused on paramedics (Murphy‐Jones and Timmons 2016). Two studies included hospital staff—one involving two emergency department physicians (Stephens et al. 2015) and another featuring a mixed focus group of nursing home, homecare and hospital nurses (Reyniers et al. 2014). However, no quotes or findings from hospital‐based staff addressed or referred to nursing home residents dying in hospitals. As such, despite screening over 12,000 studies, perspectives from hospital clinicians are notably absent from the qualitative literature.

**TABLE 1:** Summary of study characteristics (*N* = 10).

Author year country	Study aim	Study design	NH setting	Sample	Data collection and analysis
Akunor et al. 2022 USA	To describe NH staff perceptions on where end‐of‐life care should be delivered, the role of Medicare hospice care and their experiences providing end‐of‐life care to residents with advanced dementia	Qualitative descriptive analysis of secondary data	13 NH from 4 states. Each had at least 85 residents and more than 10 residents with advanced dementia	Purposive sampling of NH staff (*n* = 158) DON (*n* = 12) Nurses (*n* = 35) Nurse assistants (*n* = 23) Prescribing providers (*n* = 16) Social Workers (*n* = 14) Administrators (*n* = 12) SLP (*n* = 11) Dieticians (*n* = 10) Chaplain (*n* = 4) OT (*n* = 3) Other (*n* = 18)	Semi‐structured interviews Thematic analysis
Alcorn et al. 2020 Scotland	To explore the factors that influence hospital admission of care home residents who then died in hospital	Mixed methods	14 CHs associated with 2 teaching hospitals	Purposive sampling of CH staff and relatives of CH residents who died in hospital CH managers (*n* = 16); CH registered nurses (*n* = 6); care assistants (*n* = 4) Relatives of deceased residents (*n* = 2)	Semi‐structured interviews Thematic analysis
Economos et al. 2020 France	To explore the factors influencing the transfer of patients living in nursing homes to hospital at the end‐of‐life	Mixed methods	466 NHs: Averaged 83 residents per NH Palliative programs in 336 (72%) NHs Medical coordinator in 428 (92%) NHs Average of 6 nurses working in each NH Night shift nurse in 83 (18%) NHs	Sampling methods not described NH medical directors (*n* = 466)	Surveys with open‐ended questions Thematic analysis of open‐ended question responses
Kouyoumdjian et al. 2019 France	To explore physicians' perceptions that lead to transfers of residents, who are at the end‐of‐life, from a nursing home to an ED	Qualitative	12 NHs with an average of 77 residents per home. 3 NHs had night nurses	Purposive sampling of NH Medical directors (*n* = 12)	Semi‐structured interviews Thematic analysis
Lane and Philip 2015 Australia	To explore the views and experiences of staff from RACFs and community palliative care services on providing palliative and end‐of‐life care in RACFs	‘Qualitative methodology’	2 RACF: 30 and 90 beds for residents of varying care needs	Purposive sampling of RACF staff (*n* = 15) Community Palliative Care Services staff (*n* = 15)	Semi‐structured interviews and focus groups Thematic analysis
Murphy‐Jones and Timmons 2016 England	To explore how paramedics make decisions when asked to transport nursing home residents nearing the end of their lives	‘Phenomenological approach’	Not described	Purposive and snowball sampling of Paramedics (*n* = 6)	Semi‐structured interviews Thematic analysis
Pechová and Loučka 2023 Czech Republic	To explore factors that influence decision‐making about the hospitalization of nursing home residents that become terminal	Qualitative with subtle realism paradigm	6 NHs chosen based on differences: more than 100 beds or less than 100 beds, in the capital region or elsewhere, cities with more than 100,000 residents or less than 20,000 and homes with or without established palliative care programs	Purposive, maximum variation sampling of NH staff (*n* = 27) Registered nurses (*n* = 12) Social workers (*n* = 5) Physicians (*n* = 10)	Semi‐structured interviews Thematic analysis
Reyniers et al. 2014 Belgium	To explore the perspectives of nurses from nursing homes, home care and hospitals and family physicians concerning hospital admissions at the end‐of‐life and the circumstances in which they consider them to be justified	Not described	NH not described	Purposeful sampling for eight focus groups (*n* = 61) Three focus groups of family physicians (*n* = 39), one focus group of nursing home nurses (*n* = 7), two groups of nursing home, homecare and hospital nurses (*n* = 16)	Focus groups were audio recorded, complemented with field notes
Sopcheck and Tappen 2023 USA	To describe preferences for residents approaching the end‐of‐life if a change in condition occurs. To explore understanding of palliative and hospice care and hospital‐based acute care purposes and practices	Qualitative design	110 bed Florida NH with 58 long‐term beds and 52 short‐term beds	Purposive sampling of residents, family members and nursing home staff (*n* = 45) Long‐term residents (*n* = 13) Short‐term residents (*n* = 2) Family members (*n* = 10) NH staff (*n* = 20)	Structured interview guide with open‐ended questions
Stephens et al. 2015 USA	To describe providers' perspectives on the role that families play in the decision to transfer NH residents to the ED	Descriptive, grounded theory	NH Not described	Purposeful and convenience sampling of NH nurses (*n* = 16), primary care providers (*n* = 11), ED physicians (*n* = 2), NH administrators (*n* = 3), hospitalist (*n* = 1)	Focus groups using semi‐structured interview guides.

Abbreviations: CH, care home; DON, Director of Nursing; ED, emergency department; EOLC, end‐of‐life care; GP, general practitioner; NH, nursing home; OT, occupational therapist; RACF, residential aged care facility; SLP, speech‐language pathologist.

None of the included studies provided details on the hospital settings where residents died. Three studies did not describe the nursing home setting where residents lived (Murphy‐Jones and Timmons 2016; Reyniers et al. 2014; Stephens et al. 2015). The remaining studies described the nursing home setting according to the number of homes included and/or the number of residents living there (Akunor et al. 2022; Alcorn et al. 2020; Economos et al. 2020; Kouyoumdjian et al. 2019; Lane and Philip 2015; Pechová and Loučka 2023; Sopcheck and Tappen 2023). Other details provided on the settings included nursing homes having palliative programs (Economos et al. 2020), night shift nurses (Economos et al. 2020; Kouyoumdjian et al. 2019; Pechová and Loučka 2023) and being associated with teaching hospitals (Alcorn et al. 2020) (See Table 1).

### 3.2 Risk of Bias

The ten included studies were of mixed quality, as outlined by our CASP evaluations in Table 2. All studies included clear aims, methodologies, participant recruitment and data collection methods and a statement of findings. Five studies met all quality appraisal criteria (Lane and Philip 2015; Murphy‐Jones and Timmons 2016; Reyniers et al. 2014; Sopcheck and Tappen 2023; Stephens et al. 2015). However, the data analysis of one study (Kouyoumdjian et al. 2019) was unclear as the authors state utilizing content analysis but present the findings according to thematic analysis, and the authors of another study (Pechová and Loučka 2023) did not mention ethical considerations. Additionally, the relationship between researchers and participants and researcher bias were not discussed in three studies (Alcorn et al. 2020; Economos et al. 2020; Kouyoumdjian et al. 2019).

**TABLE 2:** CASP assessment scores for included studies (*N* = 10).

CASP criteria	Akunor et al. (2022)	Alcorn et al. (2020)	Economos et al. (2020)	Kouyoumdjian et al. (2019)	Lane and Philip (2015)	Murphy‐Jones and Timmons (2016)	Pechová and Loučka (2023)	Reyniers et al. (2014)	Sopcheck and Tappen (2023)	Stephens et al. (2015)
Was there a clear statement of the aims of the research?	Y	Y	Y	Y	Y	Y	Y	Y	Y	Y
Is a qualitative methodology appropriate?	Y	Y	Y	Y	Y	Y	Y	Y	Y	Y
Was the research design appropriate to address the aims of the research?	Y	Y	Y	Y	Y	Y	Y	Y	Y	Y
Was the recruitment strategy appropriate to the aims of the research?	Y	Y	Y	Y	Y	Y	Y	Y	Y	Y
Were the data collected in a way that addressed the research issue?	Y	Y	Y	Y	Y	Y	Y	Y	Y	Y
Has the relationship between researcher and participants been adequately considered?	N	U	N	N	Y	Y	Y	Y	Y	Y
Have ethical issues been taken into consideration?	Y	Y	Y	Y	Y	Y	N	Y	Y	Y
Was the data analysis sufficiently rigorous?	Y	Y	Y	U	Y	Y	Y	Y	Y	Y
Is there a clear statement of findings?	Y	Y	Y	Y	Y	Y	Y	Y	Y	Y
How valuable is the research?	Y	Y	Y	Y	Y	Y	Y	Y	Y	Y

*Note:* Y = criteria met; N = criteria not met; U = unclear. Colored font used to show N's and U's.

### 3.3 Findings

Through reciprocal and refutational translation across studies, two overarching interpretive themes were constructed that capture how hospitals are understood as places of death for nursing home residents: (1) Controlling symptoms of dying: comfort and discomfort; (2) Acceptance of death after all efforts to save life have been exhausted. Quotations from researchers' interpretations and direct quotations from participants in the primary studies are used to illustrate each theme and are presented as follows: ‘researcher interpretations’, ‘participant quotes’. Findings are presented narratively according to theme, with Figure 2 visually depicting the conceptual relationships across stakeholder perceptions.

**FIGURE 2:**
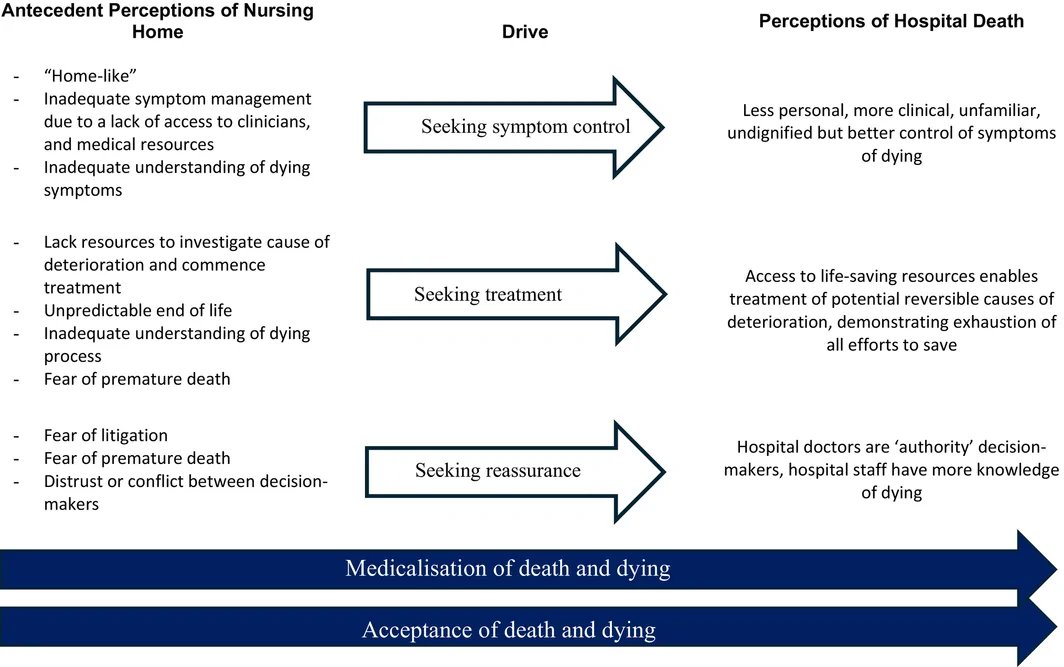
Conceptual model illustrating how antecedent perceptions of nursing homes drive hospital transfer and shape perceptions of hospital death. How fear, uncertainty and perceived inadequacy generate hospital transfer as a mechanism of control, authority and legitimized death.

Notably, no included studies reported data from hospital clinicians; most studies explored the perspectives of nursing home clinicians, paramedics, bereaved family members and residents. Table 3 details the contributions of each participant group to the coding and thematic development, including data that informed analysis but was not directly represented in the final third‐order synthesis.

**TABLE 3:** Participant contributions to first‐, second‐ and third‐order constructs across included studies (*N* = 10).

Third‐order concepts	Second‐order concepts	Concepts from first‐order quotes	Akunor et al. (2022)	Alcorn et al. (2020)	Economos et al. (2020)	Kouyoumdjian et al. (2019)	Lane and Philip (2015)	Murphy‐Jones and Timmons (2016)	Pechová and Loučka (2023)	Reyniers et al. (2014)	Sopcheck and Tappen (2023)	Stephens et al. (2015)
Controlling symptoms of dying: comfort and discomfort	Some symptoms are better managed in the hospital	Decrease suffering						Pa	F	P	R, F	
		Dehydration		M					N			
		Seizure		M					P			
		Pain		M	D	D	N		N	P	N	P
		Dyspnoea		M		D	N		N			
		Vomiting/diarrhoea/bleeding		N		D			P			
		Unpredictable deterioration		N					P, N			N
		Psychological distress/behaviour				D			N	P	N	
	Hospitals have greater access to resources and clinicians	Equipment		M	D	D	N		N, P			
		Medications		N, M	D	D			N		N	
		Physicians		M					N			P
		‘Better’ nursing care, more staff		M	D			Pa	P, SW	N, P	N, R, F	N
	Hospital may be unfamiliar, more clinical and a less comfortable place to die	Hospital unfamiliar	NP	N							F, R	
		Nursing home is like family/home; personalized, holistic care	N, D	N	D		N		N	P	N, R	
		Disappointed in death occurring at a hospital death		M			N			P		
Acceptance of death after all efforts to save life have been exhausted	Seeking treatment of potentially reversible causes of deterioration	Family wishes to transfer		M					N			N
		Importance of advance care directive		N	D				P		N	
		Difficulty in identifying dying	NP	N, F					N			
		Hospital is comforting, aids death acceptance		M, N		D	N	Pa	N	P		N
	Death acceptance: reassurance from the medical hierarchy of hospital clinicians	Uncertainty/disagreements arise		M	D				N			N
		Fear of not doing enough		M		D						N
		Fear of litigation						Pa	N			
		Lack of trust in nursing home		F			N	Pa	P	P	F	P

*Note:* D = Nursing home Director of Care; P = Nursing home Physician; N = Nursing home Nurse; F = Resident family member; Pa = Paramedic; NP = Nursing home Nurse Practitioner; R = resident; SW = Nursing home Social Worker; M = Nursing Home Manager.

#### 3.3.1 Controlling Symptoms of Dying: Comfort and Discomfort

In this theme, we explored the strong perception identified in nine studies that nursing home residents experience improved symptom management in the hospital setting due to improved access to resources including clinicians and medical equipment not available in the nursing home setting (Alcorn et al. 2020; Economos et al. 2020; Kouyoumdjian et al. 2019; Lane and Philip 2015; Murphy‐Jones and Timmons 2016; Pechová and Loučka 2023; Reyniers et al. 2014; Sopcheck and Tappen 2023; Stephens et al. 2015). This theme consists of three subthemes: (1) Some symptoms are better managed in the hospital; (2) Hospitals have greater access to medical resources and clinicians; (3) Hospitals may be unfamiliar, more clinical and a less comfortable place to die.

##### 3.3.1.1 Some Symptoms Are Better Managed in the Hospital

Suffering was described in terms of resident symptoms and most frequently included pain (Alcorn et al. 2020; Economos et al. 2020; Kouyoumdjian et al. 2019; Lane and Philip 2015; Pechová and Loučka 2023; Reyniers et al. 2014; Sopcheck and Tappen 2023; Stephens et al. 2015), acute dyspnoea (Alcorn et al. 2020; Kouyoumdjian et al. 2019; Lane and Philip 2015; Pechová and Loučka 2023) and psychological distress (Kouyoumdjian et al. 2019; Pechová and Loučka 2023; Reyniers et al. 2014; Sopcheck and Tappen 2023).

Several residents reported they would consider transferring to the hospital if the nursing home ‘could not help me’ or to promote their comfort (resident, Sopcheck and Tappen 2023), and death in a hospital was considered necessary when participants perceived all options available in the nursing home had been exhausted (Pechová and Loučka 2023). One daughter explained:

Because I would not want her to suffer here in the nursing home if the hospital could make her comfortable. (Family‐member, Sopcheck and Tappen 2023)

Pain was the most frequently identified symptom that may benefit from terminal care in the hospital:

They might be getting better pain relief and everything at the hospital. (Nurse, Lane and Philip 2015)

A nursing home nurse in Sopcheck and Tappen (2023) identified that behavioural challenges could be better managed in the hospital:

Sometimes they're extremely combative, and we can't handle them. They're not safe so … we don't want them to hurt themselves even though it's the end of life. You still need to be able to help the patient, make him comfortable, and if we cannot meet their needs here, we have to find someplace else to meet them. (Nurse, Sopcheck and Tappen 2023)

Other conditions were seen beyond the scope of what could be provided for in the nursing home:

She would remain here and been looked after but unfortunately with the dislocated jaw and things like that it was kind of out with our range kind of thing that we could manage. (Care Home Manager, Alcorn et al. 2020)

However, Kouyoumdjian et al. (2019) reported ‘Some medical directors interviewed think those refractory symptoms are, in fact, rare’ and cited one Nursing Home Director:

We can manage those situations with a few therapeutics. (Nursing Home Director)

##### 3.3.1.2 Hospitals Have Greater Access to Medical Resources and Clinicians

Six studies identified improved symptom management in the hospital setting was possible due to perceptions that hospitals have greater access to resources, such as medical equipment (Alcorn et al. 2020; Economos et al. 2020; Kouyoumdjian et al. 2019; Lane and Philip 2015; Pechová and Loučka 2023; Sopcheck and Tappen 2023). The most commonly identified equipment available in hospitals to support symptom management at the end‐of‐life included electrical syringes, oxygen, suction machines, infusion sets, non‐standard medications and access to medications (Alcorn et al. 2020; Economos et al. 2020; Kouyoumdjian et al. 2019; Lane and Philip 2015; Pechová and Loučka 2023; Sopcheck and Tappen 2023). One Nursing Home Director explained:

In a nursing home, there is not always electrical syringe, hospital products, even for palliative care […] I sent someone at end‐of‐life […] with acute dyspnoea, who was conscious, who could not breathe, but who we had no way of relieving and who left our world quietly in an emergency department […] within 24h with midazolam continuous injection. (Nursing Home Director, Kouyoumdjian et al. 2019)

Participants in seven studies perceived hospitals to have greater access to clinicians, such as 24/7 nursing presence, with more comprehensive skills to provide ‘better’ end‐of‐life care (Alcorn et al. 2020; Economos et al. 2020; Pechová and Loučka 2023; Reyniers et al. 2014; Sopcheck and Tappen 2023; Stephens et al. 2015). One resident reported preferring to die in the hospital due to the nursing staff:

Well, they've had more training. Well, they have real nurses. (Resident, Sopcheck and Tappen 2023)

Another resident explained their perceptions of hospital as a place of death compared with the nursing home:

You don't get that much care. You get it as much as they could do. In the hospital sometimes, it's a little better … (Resident, Sopcheck and Tappen 2023)

A family member also perceived some potential benefits of end‐of‐life care in hospital:

If the transferring of hospital was necessary, it would be for the more one on one care … that would make her comfortable. (Family‐member, Sopcheck and Tappen 2023)

Some participants also perceived hospitals to have improved access to physicians compared to nursing homes:

… there's no priority given to care homes, erm, our phone calls go into the same waiting list as anybody else's… … it could be 10 hours. (Nursing Home Manager, Alcorn et al. 2020)

##### 3.3.1.3 Hospitals May Be Unfamiliar, More Clinical and a Less Comfortable Place to Die

The hospital environment and care culture were perceived to be less holistic compared to nursing homes (Akunor et al. 2022; Alcorn et al. 2020; Economos et al. 2020; Lane and Philip 2015; Pechová and Loučka 2023; Reyniers et al. 2014; Sopcheck and Tappen 2023). A nursing home nurse compared the nursing home with the hospital as ‘less clinical…[and]…not as cold’ (Nursing Home Nurse, Akunor et al. 2022), which Akunor et al. (2022) interpreted as nursing homes having the capacity to provide ‘more personalized’ and ‘holistic’ care ‘focused on the whole person’ (Researchers' Interpretation, Akunor et al. 2022).

It was reported that in the nursing home, residents would ‘have people that love them and take care of them and have that bond with them that people in the hospital doesn't have’ (Nursing Home Nurse, Alcorn et al. 2020). Conversely, hospitals were described as ‘unfamiliar, undignified places to die’ (Nursing Home Director, Alcorn et al. 2020), ‘focusing on your disease’ (Nursing Home Director, Akunor et al. 2022) and ‘somewhere where people don't know you’ (Nursing Home Administrator, Akunor et al. 2022). One nursing home manager stated:

I don't know anybody that wants to die in hospital. Especially people with dementia… the staff are familiar, they've got their room, everything, … I think to put them in an acute, sort of medical ward and to have to die there is very undignified and I think it must be hard for the families as well. (Care Home Manager, Alcorn et al. 2020)

#### 3.3.2 Acceptance of Death After All Efforts to Save Life Have Been Exhausted

In this theme, we describe how many clinicians and family members experienced greater acceptance of death after residents had accessed hospital services and were seen by a hospital clinician. This theme contains two subthemes: (1) Seeking treatment of potentially reversible causes of deterioration; (2) Acceptance of death: reassurance and the medical hierarchy of hospital clinicians.

##### 3.3.2.1 Seeking Treatment of Potentially Reversible Causes of Deterioration

The hospital was perceived to be a good place to die as a demonstration that all efforts to save life had been exhausted. In seven studies, nursing home nurses, physicians and directors and paramedics expressed finding comfort and reassurance knowing the resident had received access to hospital services that could have prolonged their life (Alcorn et al. 2020; Kouyoumdjian et al. 2019; Lane and Philip 2015; Murphy‐Jones and Timmons 2016; Pechová and Loučka 2023; Reyniers et al. 2014; Stephens et al. 2015). In several cases, nursing home staff perceived that hospitals would engage in ‘establishing a diagnosis and instituting curative treatment’ with an ‘expectation that the resident would return’ (Researchers' Interpretations, Alcorn et al. 2020). Another nurse reflected on a resident who had died in hospital: ‘we wanted to save their life’ (Nursing Home Nurse, Lane and Philip 2015).

It was evident that the option of trialling treatment in hospital sometimes usurped residents' preference to remain in the nursing home. A daughter of a resident who died in hospital explained:

She [nursing home nurse] phoned to say he's really struggling…and I could hear my dad saying “no”, so I said “no he doesn't want that, can we not get the respiratory team?” and I think she tried … in the end they'd actually just called the ambulance because his oxygen levels were so low … by that stage he was resigned to going back into hospital so we just let him go. (Family‐member, Alcorn et al. 2020)

The nursing home nurse caring for this resident reported:

We know that he doesn't want to go into hospital, that was his wishes, not to [go to hospital], but we needed to do something, so we called an ambulance. (Nursing Home Nurse, Alcorn et al. 2020)

Similarly, a care home manager described overriding a resident's preference to remain in the nursing home as it was perceived they may benefit from life‐prolonging treatment:

I remember the day she was admitted into hospital she said ‘you're making me go’, I said ‘you really need to go’. I think she had to get IV antibiotics and we couldn't, the orals just weren't touching her here. So that was really quite sad… because you always expect they're going to come back. (Care Home Manager, Alcorn et al. 2020)

Nursing home clinicians often perceived that family members and residents had greater acceptance of death if the resident was transferred to a hospital for further treatment, even if they died while there. For example, a nursing home manager explains:

The plan was she wasn't going to go to hospital, it would just be a peaceful, you know, keep her here where she knew everybody but she took such a massive deterioration very quickly overnight and the family decided no they wanted her to be treated, and that's where we got into difficulty because everybody then started to go ‘oh, we don't think she's going to even make the journey’…it was very traumatic. (Nursing Home Manager, Alcorn et al. 2020)

In some cases, nursing home clinicians reported they had explained the futility of pursuing life‐prolonging treatment but honoured the resident or family‐member's wishes for hospital transfer:

Almost all [nursing home nurses and physicians] agreed that when anxiety or false hope cannot be taken away by providing more information and the patient persistently requests to be transferred to hospital for further care or to die, their autonomy in this regard should not be questioned. (Researchers' Interpretation, Reyniers et al. 2014)

However, nursing home clinicians perceived death in hospital as unacceptable if the death occurred as an unintended outcome of a hospital transfer (Akunor et al. 2022; Alcorn et al. 2020; Pechová and Loučka 2023), highlighting the difficulty nursing home clinicians had in identifying residents at the end of their lives. The ‘inherent unpredictability in residents' decline’ and ‘uncertain dwindling trajectories, multimorbidity including cognitive impairment and acute deteriorations in residents’ can make recognizing dying challenging (Researchers' Interpretations, Alcorn et al. 2020; Pechová and Loučka 2023). Pechová and Loučka (2023) reported that ‘over half of the hospitalizations were not anticipated as terminal, [they] were qualified as sudden, unexpected, and not anticipated’ (Researchers' Interpretation). A care home manager reflected:

It was a complete surprise because that was not expected at all, you know, we were really upset when Sarah passed away… she wasn't end‐of‐life by any, we were completely, completely dumbfounded. (Care Home Manager, Alcorn et al. 2020)

In several instances, there was a perception that nursing home nurses did not think that dying in a hospital was best for the resident and many felt they were not able to deliver an expected duty of providing end‐of‐life care: ‘it goes against our life's work’ (Nursing Home Director, Economos et al. 2020).

##### 3.3.2.2 Death Acceptance: Reassurance From the Medical Hierarchy of Hospital Clinicians

There was a strong perception of hospitals being the preferred place of death when there was uncertainty or conflict between nursing home decision‐makers (e.g., nurses and family members) or a lack of trust in the nursing home (Alcorn et al. 2020; Economos et al. 2020; Lane and Philip 2015; Murphy‐Jones and Timmons 2016; Pechová and Loučka 2023; Reyniers et al. 2014; Stephens et al. 2015). For example, when reflecting on a resident dying in a hospital, one nursing home nurse stated:

“…she had a son and a daughter, and they were both responsible party. So the son told me, ‘oh, it's DNR, keep her there, keep her comfortable’. Okay, so I'm going to follow that…and then I got a call from the doctor who said if they want her out [to the ED], go ahead and send her out. The daughter comes running in, ‘oh, send her now’. I was like your brother said – ‘don't listen to him’ (said the daughter)” (Nursing Home Nurse, Stephens et al. 2015).

Nursing home staff and paramedics perceived that nursing home residents dying in hospital reduced their burden of professional risk, mitigating the potential for legal proceedings if death was not accepted by family members. For example, when nursing home clinicians felt professionally vulnerable, they preferred for residents to die in hospital where the responsibility for end‐of‐life care was absolved to hospital clinicians. A paramedic reported overhearing nurses say ‘I can't take the risk’ [keeping the resident at the nursing home] and another paramedic noted:

The person who is incapacitated is not going to complain about your treatment but their family may well do. (Paramedic, Murphy‐Jones and Timmons 2016)

After sending a terminally ill resident to hospital, a nurse reported:

I just called because I know how the family is. And I do not want to go to court. (Nursing Home Nurse, Pechová and Loučka 2023)

Similarly, the family relationship with and degree of trust in nursing home clinicians influenced their preferences for a resident's place of death when considering end‐of‐life decision‐making and end‐of‐life care. One family member explained:

I think I would trust the hospital a little bit more than… the nursing home. (Family‐member, Sopcheck and Tappen 2023)

## 4 Discussion

In this meta‐ethnography, we synthesized the evidence about nursing home residents' deaths in hospitals. Ten studies from seven countries were included. Data were primarily sourced from nursing home staff perceptions (Akunor et al. 2022; Alcorn et al. 2020; Economos et al. 2020; Kouyoumdjian et al. 2019; Lane and Philip 2015; Pechová and Loučka 2023; Reyniers et al. 2014), with some insight from paramedics (Murphy‐Jones and Timmons 2016) and bereaved family members of residents who had died in a hospital (Alcorn et al. 2020; Sopcheck and Tappen 2023). We identified a dearth of research exploring hospital clinicians' perceptions of nursing home resident deaths in hospital. Further insight is needed regarding hospital clinicians' perceptions of their role and capacity to provide end‐of‐life care in a setting traditionally focused on acute, life‐saving interventions.

Our third‐order synthesis suggests that hospitals occupy a paradoxical position (Figure 2): they are simultaneously perceived as impersonal and clinically oriented environments, and as resource‐rich sites capable of alleviating suffering and legitimizing death through medical authority.

### 4.1 Hospital Death and the Alleviation of Suffering

Consistent with other reviews, our meta‐synthesis identified that hospitals were perceived to be impersonal, clinically focused and unfamiliar settings for end‐of‐life care (Moon et al. 2018; Pinto et al. 2024; Yamout et al. 2022). Despite these discomforts, the findings from this meta‐ethnography and a previous umbrella review revealed strong perceptions that patients receive better symptom management in hospitals (Pinto et al. 2024). While these perceptions suggest hospitals are believed to provide superior symptom management, evidence regarding the quality of end‐of‐life care in hospital settings remains mixed. For example, a systematic review exploring end‐of‐life care for older adults with dementia who died in hospital identified that family members and healthcare practitioners perceived pain and anxiety were prevalent in death and that end‐of‐life assessments were inadequately documented (Moon et al. 2018).

Perceptions that hospitals will alleviate suffering and provide symptom control may stem from attempts to control the dying process in ways that comfort family members and healthcare practitioners (Cottrell and Duggleby 2016; Sallnow et al. 2022). Being free of the physical symptoms of dying, especially pain, is often perceived as the hallmark of a good death (Cottrell and Duggleby 2016; Ijaopo et al. 2023; Zaman et al. 2021). Yet, dying can often look uncomfortable and family members and nursing home nurses are frequently unprepared for it, sometimes missing or misinterpreting the signs of imminent death (Bagchus et al. 2024). A study exploring caregivers' preparedness for death found that perceptions of reduced suffering in the hospital setting could be due to lack of education about end‐of‐life and preparedness for death (Durepos et al. 2020). In their systematic review of systematic reviews into the conditions of a good death, Zaman et al. (2021) identified that most medical interventions did not require hospitalization, emphasizing the need to downplay medicine's role at the end of life. Sallnow et al. (2022) highlight that death has become increasingly medicalized, shifting care of the dying from being a family event with little medical intervention to a medical event with little family involvement.

### 4.2 Hospitalization, Medicalization and Death Acceptance

In our meta‐synthesis, we identified that a preference for hospital death was profoundly influenced by nursing home clinicians' and family members' fears of premature death, at times usurping the resident's preference to remain in the nursing home. A hospital death was perceived to demonstrate that all opportunities to treat any reversible causes of deterioration had been exhausted. These findings are echoed in an umbrella review of patient' and family members' preferences for place of death, which identified that hospital care at the end‐of‐life were associated with a perception of greatest chance of survival (Pinto et al. 2024).

Our findings and those of previous studies highlight that nursing home clinicians continue to experience fears of being accused of ‘failure to act’ and the potential for litigation if family members perceive their loved one died prematurely (Laging et al. 2015). While our meta‐synthesis did not provide data from the perspectives of hospital clinicians' end‐of‐life decision‐making, a scoping review exploring clinical reasoning in emergency medical practice identified physician's experience fear of litigation and concerns about malpractice, contributing to an increased propensity for ordering investigations and admitting patients to hospital (Pelaccia et al. 2020).

Taken together, these findings suggest that the roles and rituals surrounding dying and death may be highly influenced by broader societal perceptions of dying being associated with a medical event rather than as a natural process. These perceptions may perpetuate the role of health services as ‘custodians of death’ (Sallnow et al. 2022) and expectations that clinicians demonstrate exhausting all possible medical interventions before transitioning to end‐of‐life care. In this way, hospital transfer functions symbolically—not only as a clinical intervention, but as a ritual that legitimizes the transition from life‐prolonging treatment to acceptance of death.

Timely recognition of dying is important for high‐quality end‐of‐life care to enable the focus of care to be adjusted so that futile and potentially burdensome interventions are avoided (Bagchus et al. 2024; Motamedi et al. 2021; Polak et al. 2020; Zaman et al. 2021). Early signs of dying are complex and multidimensional, encompassing physical, psychological and spiritual patient dimensions, such as low mood, lack of interest and increased confusion (Bagchus et al. 2024), and as such, the early signs of dying are missed until very close to the time of death (Persson et al. 2018). No studies in our review reported hospital clinicians' perspectives on recognizing and responding to end‐of‐life care needs; however, a previous review of older people with dementia who died in hospital revealed inconsistent approaches to aggressive and invasive treatment (Moon et al. 2018). A scoping review exploring perceptions of end‐of‐life care among older patients and informal caregivers identified that a lack of clear communication led families to pursue curative treatments of older adults as they neared their end‐of‐life (Motamedi et al. 2021). Such findings highlight that both hospital clinicians and nursing home clinicians experience challenges recognizing and responding to end‐of‐life care needs of older people.

### 4.3 Recommendations

Supporting residents to die in their preferred place of death is a key priority. Several studies have reported residents' preferences to die in the nursing home (Greenwood et al. 2018; Moberley et al. 2023; Ng et al. 2016; Searle et al. 2023). For example, the authors from one retrospective medical record review of 234 deceased nursing home residents reported that ‘Most residents (96.7%) wished to remain in their residential aged care facility if their health deteriorated in an expected way’ (Moberley et al. 2023). These findings highlight the importance of addressing the factors contributing to the marginalization of resident preferences to receive end‐of‐life care in the nursing home.

A critical success factor is the need for greater recognition across societal, policy and practice levels that deterioration, dying and death are expected, impending and natural processes that all frail older people in nursing homes will experience. Changes to health policies and quality standards to embrace dying and death as an expected outcome are critical to supporting the role of nursing homes as providers of end‐of‐life care and embedding consistent provision of palliative care resources across the sector. This includes greater consideration of the additional supports that are needed to reduce nursing home clinicians' perceptions of professional vulnerability when they engage in decisions to transition a resident to end‐of‐life care.

Defining dying as a holistic process, rather than a medical event may increase acceptance of nursing home end‐of‐life care provision. To support this, nursing home residents and family members will benefit from greater opportunities to engage in holistic end‐of‐life care planning. This could include expanding advanced care planning beyond medicalized discussions associated with the benefits and limitations of specific treatments to incorporate greater education about the dying process and the benefits and limitations of interventions to support end‐of‐life care.

It is important to recognize the value of providing timely access to medical resources to support end‐of‐life care in the nursing home setting. For instance, previous reviews have reported a reduction in resident's dying in hospital when nursing home resident's access to nurse practitioners or clinical nurse specialists (Allers et al. 2019), additional end‐of‐life care services (Buck et al. 2022), a palliative care team (Searle et al. 2023) and higher numbers of registered nurses (Searle et al. 2023). Additionally, a scoping review of telehealth palliative care in nursing homes identified consistent benefits associated with telehealth access to specialist palliative care clinicians (physicians or nurse practitioners), including increased documentation of goals of care and decreased hospitalizations (Walton et al. 2023). However, palliative care and telehealth services are inconsistently available to nursing homes. There is also inadequate understanding of the prescribing, access, storage, use and disposal of controlled drugs for end‐of‐life care across nursing homes (Majumder et al. 2022).

Greater oversight and benchmarking of end‐of‐life resourcing in nursing homes is needed to ensure consistent access to high‐quality end‐of‐life care. Variability in staffing, training and palliative support may perpetuate confusion and distrust among residents and family members regarding the capacity of nursing homes to provide appropriate end‐of‐life care. Our review found that although death in hospital was often described as undesirable, it continues to occur, at times driven by perceptions that adequate care cannot be delivered within the nursing home setting. Strengthening palliative care within nursing homes, alongside efforts to improve confidence in their ability to provide end‐of‐life care, is essential to reducing potentially avoidable hospital deaths.

Importantly, our findings do not suggest that hospital deaths are inherently inappropriate, but rather that decisions surrounding hospital transfer are shaped by structural constraints, professional anxieties and cultural expectations about medical authority. Nevertheless, as the global population continues to age, residents will likely continue to present at hospitals at the end‐of‐life. Therefore, greater consideration must be given to the hospital environment to support the end‐of‐life care experience for nursing home residents.

### 4.4 Recommendations for Research

In our meta‐synthesis, we identified widespread perceptions that dying in hospital may be associated with improved symptom management. However, we were not able to determine whether hospital clinicians' perceived improvements in symptom management when compared with nursing homes, highlighting an important gap in knowledge and opportunities for further research. Greater understanding of the presenting symptoms associated with terminal hospitalization from nursing homes may support focused interventions to support nursing home end‐of‐life care.

Future research is needed to explore perceptions of suffering at the end‐of‐life and how these shape preferences for place of death. We identified negative views of the hospital setting and its perceived impact on reducing the quality of end‐of‐life experience. Accordingly, further research should examine how hospital environmental design adaptations might support a more private and less clinical experience for dying individuals and their families. Further research is also needed to improve our understanding of how timely access to palliative care services may contribute to improving quality of care by reducing aggressive medical interventions at the end‐of‐life and concomitantly reducing costs (Luta et al. 2021; Naoum et al. 2023).

### 4.5 Strengths and Limitations

The strength of this meta‐ethnography lies in the three stages of analysis, allowing the researchers to go beyond aggregation of the descriptions of the perceptions of hospital as a place of death and to extrapolate the underlying drivers of these perceptions. We also acknowledge that consensus regarding the perceived role of hospitals in the care of nursing home residents cannot be conclusive and is rather an individually and circumstantially constructed perspective (Sattar et al. 2021). The findings from synthesis are also limited to the data presented in each paper as the reviewers did not have access to the original data within each study.

Despite the large spread of countries included in our review, the research was predominantly undertaken in high‐income countries from the perspectives of nursing home clinicians, paramedics and bereaved family members. Furthermore, those who have died in a hospital cannot report on their perceptions and therefore our understanding from the residents' perspective is limited to their preconceptions of hospital death. We recognize that the practices and beliefs of healthcare professionals and consumers of end‐of‐life care are framed by culture and religious and spiritual beliefs (Zaman et al. 2021). Further understanding can be generated through purposeful sampling of hospital clinicians' and wider cultural perspectives. Less experienced healthcare professionals may also report greater levels of discomfort providing end‐of‐life care and it is therefore important that future studies consider the experience levels of clinicians and how this may have influenced their perceptions (Croxon et al. 2018; Karbasi et al. 2018). Despite these limitations, the perceptions of hospital death from the perspective of nursing home clinicians, residents and family members provide key insights into the drivers of hospital transfer and opportunities to strengthen health service provision for older people at the end‐of‐life across all health settings.

## 5 Conclusion

Strong perceptions persist that hospitals offer a safer setting for end‐of‐life care because they provide ready access to medical resources and clinicians who can manage symptoms effectively. Hospitals are also viewed as places where investigations can be conducted and any potentially reversible causes of deterioration can be treated, helping to alleviate fears of a premature death. Supporting residents' preferences to die in nursing homes requires earlier, more holistic engagement with the realities of dying, alongside equitable access to palliative care resources across care settings.

## Author Contributions

Bridget Laging: conceptualization, methodology, formal analysis, writing – original draft, writing – review and editing, visualization, supervision, project administration, funding acquisition. Rachel MacLean: methodology, formal analysis, writing – original draft, writing – review and editing, visualization. Jessica Dickson: resources, writing – review and editing. Janine Alan: conceptualization, writing – review and editing.

## Funding

This work was supported by the Equity Trustees, 2024. The funder had no involvement in the completion of this study.

## Conflicts of Interest

The authors declare no conflicts of interest.

## Supporting information


**Table S1:** eMERGe reporting statement.
**Table S2:** Search strategy for MEDLINE, CINAHL, PsycINFO and Scopus.

## Data Availability

The data that support the findings of this study are available from the corresponding author upon reasonable request.

## References

[opn70101-bib-0001] Akunor, H. S. , E. P. McCarthy , M. Hendricksen , et al. 2022. “Nursing Home Staff Perceptions of End‐of‐Life Care for Residents With Advanced Dementia: A Multisite Qualitative Study.” Journal of Hospice and Palliative Nursing 24, no. 3: 152–158. 10.1097/NJH.0000000000000843. PMC9058147 35195109

[opn70101-bib-0002] Alcorn, G. , S. A. Murray , and J. Hockley . 2020. “Care Home Residents Who Die in Hospital: Exploring Factors, Processes and Experiences.” Age and Ageing 49, no. 3: 468–480. 10.1093/ageing/afz174. 32091569

[opn70101-bib-0003] Allers, K. , F. Hoffmann , and R. Schnakenberg . 2019. “Hospitalizations of Nursing Home Residents at the End of Life: A Systematic Review.” Palliative Medicine 33, no. 10: 1282–1298. 10.1177/0269216319866648. PMC6899437 31368855

[opn70101-bib-0004] Bagchus, C. , M. Zee , J. van der Steen , et al. 2024. “Challenges in Recognizing and Discussing Changes in a Resident's Condition in the Palliative Phase: Focus Group Discussions With Nursing Staff Working in Nursing Homes About Their Experiences.” BMC Palliative Care 23, no. 1: 144. 10.1186/s12904-024-01479-3. PMC11163817 38858719

[opn70101-bib-0005] Buck, D. , S. Tucker , B. Roe , J. Hughes , and D. Challis . 2022. “Hospital Admissions and Place of Death of Residents of Care Homes Receiving Specialist Healthcare Services: A Systematic Review Without Meta‐Analysis.” Journal of Advanced Nursing 78, no. 3: 666–697. 10.1111/jan.15043. 34532884

[opn70101-bib-0006] Cable‐Williams, B. , and D. M. Wilson . 2017. “Dying and Death Within the Culture of Long‐Term Care Facilities in Canada.” International Journal of Older People Nursing 12, no. 1: e12125. 10.1111/opn.12125. 27431427

[opn70101-bib-0007] Cardona‐Morrell, M. , J. C. Kim , M. Brabrand , B. Gallego‐Luxan , and K. Hillman . 2017. “What Is Inappropriate Hospital Use for Elderly People Near the End of Life? A Systematic Review.” European Journal of Internal Medicine 42: 39–50. 10.1016/j.ejim.2017.04.014. 28502866

[opn70101-bib-0008] Clark, D. 2002. “Between Hope and Acceptance: The Medicalisation of Dying.” British Medical Journal 324, no. 7342: 905–907. 10.1136/bmj.324.7342.905. PMC1122840 11950744

[opn70101-bib-0009] Costa, V. , C. C. Earle , M. J. Esplen , et al. 2016. “The Determinants of Home and Nursing Home Death: A Systematic Review and Meta‐Analysis [Article].” BMC Palliative Care 15: 8. 10.1186/s12904-016-0077-8. PMC4721064 26791258

[opn70101-bib-0010] Cottrell, L. , and W. Duggleby . 2016. “The “Good Death”: An Integrative Literature Review.” Palliative & Supportive Care 14, no. 6: 686–712. 10.1017/S1478951515001285. 26732508

[opn70101-bib-0011] Critical Appraisal Skills Programme, C . 2023. “CASP Qualitative Research Checklist.” https://casp‐uk.net/casp‐tools‐checklists/.

[opn70101-bib-0012] Croxon, L. , L. Deravin , and J. Anderson . 2018. “Dealing With End of Life‐New Graduated Nurse Experiences.” Journal of Clinical Nursing 27, no. 1–2: 337–344. 10.1111/jocn.13907. 28557177

[opn70101-bib-0013] Durepos, P. , J. Ploeg , N. Akhtar‐Danesh , T. Sussman , E. Orr , and S. Kaasalainen . 2020. “Caregiver Preparedness for Death in Dementia: An Evaluation of Existing Tools.” Aging & Mental Health 24, no. 10: 1671–1680. 10.1080/13607863.2019.1622074. 31144986

[opn70101-bib-0015] Economos, G. , F. Tholly , W. Rhondali , et al. 2020. “Nursing Home Hospital Transfers in the Terminally Ill: Night Shift Nurses Matter!” BMJ Supportive & Palliative Care 10, no. 2: 228–233. 10.1136/bmjspcare-2019-001832. 31530555

[opn70101-bib-0016] France, E. F. , M. Cunningham , N. Ring , et al. 2019. “Improving Reporting of Meta‐Ethnography: The eMERGe Reporting Guidance.” BMC Medical Research Methodology 19: 1–13. 10.1186/s12874-018-0600-0. PMC6359764 30709371

[opn70101-bib-0017] Greenwood, N. , E. Menzies‐Gow , D. Nilsson , D. Aubrey , C. L. Emery , and A. Richardson . 2018. “Experiences of Older People Dying in Nursing Homes: A Narrative Systematic Review of Qualitative Studies.” BMJ Open 8, no. 6: e021285. 10.1136/bmjopen-2017-021285. PMC5988179 29866732

[opn70101-bib-0018] Hoffmann, F. , and K. Allers . 2020. “Ten‐Year Trends in End‐of‐Life Hospitalizations of Nursing Home Residents in Germany, 2006–2015.” Maturitas 134: 47–53. 10.1016/j.maturitas.2020.02.006. 32143776

[opn70101-bib-0019] Huffman, J. L. , and B. Harmer . 2024. “End‐Of‐Life Care.” In StatPearls. StatPearls Publishing. 31334996

[opn70101-bib-0020] Ijaopo, E. O. , K. M. Zaw , R. O. Ijaopo , and M. Khawand‐Azoulai . 2023. “A Review of Clinical Signs and Symptoms of Imminent End‐Of‐Life in Individuals With Advanced Illness.” Gerontology & Geriatric Medicine 9: 23337214231183243. 10.1177/23337214231183243. PMC10327414 37426771

[opn70101-bib-0021] Karbasi, C. , E. Pacheco , C. Bull , A. Evanson , and W. Chaboyer . 2018. “Registered Nurses' Provision of End‐of‐Life Care to Hospitalised Adults: A Mixed Studies Review.” Nurse Education Today 71: 60–74. 10.1016/j.nedt.2018.09.007. 30245257

[opn70101-bib-0022] Kouyoumdjian, V. , E. Perceau‐Chambard , C. Sisoix , M. Filbet , and C. Tricou . 2019. “Physician's Perception Leading to the Transfer of a Dying Nursing Home Resident to an Emergency Department: A French Qualitative Study.” Geriatrics and Gerontology International 19, no. 3: 249–253. 10.1111/ggi.13600. 30623550

[opn70101-bib-0023] Laging, B. , R. Ford , M. Bauer , and R. Nay . 2015. “A Meta‐Synthesis of Factors Influencing Nursing Home Staff Decisions to Transfer Residents to Hospital.” Journal of Advanced Nursing 71, no. 10: 2224–2236. 10.1111/jan.12652. 25818343

[opn70101-bib-0024] Laging, B. , A. Kenny , M. Bauer , and R. Nay . 2018. “Recognition and Assessment of Resident'deterioration in the Nursing Home Setting: A Critical Ethnography.” Journal of Clinical Nursing 27, no. 7–8: 1452–1463. 10.1111/jocn.14292. 29396884

[opn70101-bib-0026] Lane, H. , and J. Philip . 2015. “Managing Expectations: Providing Palliative Care in Aged Care Facilities.” Australasian Journal on Ageing 34, no. 2: 76–81. 10.1111/ajag.12085. 24521162

[opn70101-bib-0027] Lemoyne, S. E. , H. H. Herbots , D. De Blick , R. Remmen , K. G. Monsieurs , and P. Van Bogaert . 2019. “Appropriateness of Transferring Nursing Home Residents to Emergency Departments: A Systematic Review.” BMC Geriatrics 19, no. 1: 17. 10.1186/s12877-019-1028-z. PMC6341611 30665362

[opn70101-bib-0028] Lofland, L. H. 2019. The Craft of Dying: The Modern Face of Death. MIT Press.

[opn70101-bib-0029] Luta, X. , B. Ottino , P. Hall , et al. 2021. “Evidence on the Economic Value of End‐of‐Life and Palliative Care Interventions: A Narrative Review of Reviews.” BMC Palliative Care 20, no. 89: 89. 10.1186/s12904-021-00782-7. PMC8223342 34162377

[opn70101-bib-0030] Majumder, M. , B. Bowers , K. Pollock , C. Goodman , I. Kuhn , and S. Barclay . 2022. “End of Life Care in UK Care Homes – Controlled Drugs: Systematic Review and Narrative Synthesis.” BMJ Supportive & Palliative Care 12, no. 3: 253. 10.1136/spcare-2021-003470. 35768201

[opn70101-bib-0031] Moberley, S. , J. Hewitt , J. Attia , et al. 2023. “Preferences and End of Life Care for Residents of Aged Care Facilities: A Mixed Methods Study.” BMC Palliative Care 22, no. 1: 124. 10.1186/s12904-023-01239-9. PMC10472708 37658403

[opn70101-bib-0032] Moon, F. , F. McDermott , and D. Kissane . 2018. “Systematic Review for the Quality of End‐Of‐Life Care for Patients With Dementia in the Hospital Setting.” American Journal of Hospice and Palliative Medicine 35, no. 12: 1572–1583. 10.1177/1049909118776985. 29871500

[opn70101-bib-0033] Motamedi, M. , C. Brandenburg , M. Bakhit , et al. 2021. “Concerns and Potential Improvements in End‐Of‐Life Care From the Perspectives of Older Patients and Informal Caregivers: A Scoping Review.” BMC Geriatrics 21: 1–12. 10.1186/s12877-021-02680-2. PMC8690959 34930177

[opn70101-bib-0034] Murphy‐Jones, G. , and S. Timmons . 2016. “Paramedics' Experiences of End‐of‐Life Care Decision Making With Regard to Nursing Home Residents: An Exploration of Influential Issues and Factors.” Emergency Medicine Journal 33, no. 10: 722–726. 10.1136/emermed-2015-205405. 27189852

[opn70101-bib-0035] Naoum, P. , K. Athanasakis , and E. Pavi . 2023. “Is Palliative Care Cost‐Effective? A Systematic Review of the Literature.” Home Health Care Management & Practice 36, no. 3: 205–216. 10.1177/10848223231209316.

[opn70101-bib-0036] Ng, C. W. L. , S. Cheong , A. Govinda Raj , W. Teo , and I. Leong . 2016. “End‐of‐Life Care Preferences of Nursing Home Residents: Results of a Cross‐Sectional Study.” Palliative Medicine 30, no. 9: 843–853. 10.1177/0269216316634242. 26962065

[opn70101-bib-0037] Noblit, G. W. , and R. D. Hare . 1999. “Chapter 5: Meta‐Ethnography: Synthesizing Qualitative Studies.” Counterpoints 44: 93–123. http://www.jstor.org/stable/42975557.

[opn70101-bib-0038] Page, M. J. , J. E. McKenzie , P. M. Bossuyt , et al. 2021. “The PRISMA 2020 Statement: An Updated Guideline for Reporting Systematic Reviews.” BMJ (Clinical Research ed.) 372: n71. 10.1136/bmj.n71. PMC8005924 33782057

[opn70101-bib-0039] Pechová, K. , and M. Loučka . 2023. “Factors Influencing Decision Making in Terminal Hospitalization of Nursing Home Residents: A Qualitative Study of the Perspective of Nurses, Social Workers, and General Practitioners.” Journal of Gerontological Social Work 66, no. 7: 942–959. 10.1080/01634372.2023.2199796. 37051657

[opn70101-bib-0040] Pelaccia, T. , L. H. Plotnick , M. C. Audétat , et al. 2020. “A Scoping Review of Physicians' Clinical Reasoning in Emergency Departments.” Annals of Emergency Medicine 75, no. 2: 206–217. 10.1016/j.annemergmed.2019.06.023. 31474478

[opn70101-bib-0041] Persson, H. A. , A. Sandgren , C.‐J. Fürst , G. Ahlström , and L. Behm . 2018. “Early and Late Signs That Precede Dying Among Older Persons in Nursing Homes: The Multidisciplinary Team's Perspective.” BMC Geriatrics 18: 1–11. 10.1186/s12877-018-0825-0. PMC6000966 29898674

[opn70101-bib-0042] Pinto, S. , S. Lopes , A. B. de Sousa , M. Delalibera , and B. Gomes . 2024. “Patient and Family Preferences About Place of End‐Of‐Life Care and Death: An Umbrella Review.” Journal of Pain and Symptom Management 67, no. 5: e439–e452. 10.1016/j.jpainsymman.2024.01.014. 38237790

[opn70101-bib-0043] Polak, L. , S. Hopkins , S. Barclay , and S. Hoare . 2020. “The Difference an End‐of‐Life Diagnosis Makes: Qualitative Interviews With Providers of Community Health Care for Frail Older People.” British Journal of General Practice 70, no. 699: e757–e764. 10.3399/bjgp20X712805. PMC7510843 32958536

[opn70101-bib-0044] Reyniers, T. , D. Houttekier , J. Cohen , H. R. Pasman , and L. Deliens . 2014. “What Justifies a Hospital Admission at the End of Life? A Focus Group Study on Perspectives of Family Physicians and Nurses.” Palliative Medicine 28, no. 7: 941–948. 10.1177/0269216314522317. 24534726

[opn70101-bib-0045] Sallnow, L. , R. Smith , S. H. Ahmedzai , et al. 2022. “Report of the Lancet Commission on the Value of Death: Bringing Death Back Into Life.” Lancet 399, no. 10327: 837–884. 10.1016/s0140-6736(21)02314-x. PMC8803389 35114146

[opn70101-bib-0046] Sattar, R. , R. Lawton , M. Panagioti , and J. Johnson . 2021. “Meta‐Ethnography in Healthcare Research: A Guide to Using a Meta‐Ethnographic Approach for Literature Synthesis.” BMC Health Services Research 21, no. 1: 50. 10.1186/s12913-020-06049-w. PMC7796630 33419430

[opn70101-bib-0047] Searle, B. , R. O. Barker , D. Stow , G. F. Spiers , F. Pearson , and B. Hanratty . 2023. “Which Interventions Are Effective at Decreasing or Increasing Emergency Department Attendances or Hospital Admissions From Long‐Term Care Facilities? A Systematic Review.” BMJ Open 13, no. 2: e064914. 10.1136/bmjopen-2022-064914. PMC9896242 36731926

[opn70101-bib-0048] Sopcheck, J. , and R. M. Tappen . 2023. “Nursing Home Resident, Family, and Staff Perspectives on Hospital Transfers for End‐of‐Life Care.” Omega 86, no. 3: 1046–1068. 10.1177/0030222821997708. 33632028

[opn70101-bib-0049] Stephens, C. , E. Halifax , N. Bui , et al. 2015. “Provider Perspectives on the Influence of Family on Nursing Home Resident Transfers to the Emergency Department: Crises at the End of Life.” Current Gerontology and Geriatrics Research 2015: 1–10. 10.1155/2015/893062. PMC4561315 26379704

[opn70101-bib-0050] Walton, L. , K. Courtright , G. Demiris , E. F. Gorman , A. Jackson , and J. G. Carpenter . 2023. “Telehealth Palliative Care in Nursing Homes: A Scoping Review.” Journal of the American Medical Directors Association 24, no. 3: 356–367.e352. 10.1016/j.jamda.2023.01.004. PMC9985816 36758619

[opn70101-bib-0051] World Health Organization . 2020. “Palliative Care.” https://www.who.int/news‐room/fact‐sheets/detail/palliative‐care.

[opn70101-bib-0052] Yamout, R. , J. Hanna , R. El Asmar , H. Beydoun , M. Rahm , and H. Osman . 2022. “Preferred Place of Death for Patients With Terminal Illness: A Literature Review.” Progress in Palliative Care 30, no. 2: 101–110. 10.1080/09699260.2021.1961985.

[opn70101-bib-0053] Zaman, M. , S. Espinal‐Arango , A. Mohapatra , and A. R. Jadad . 2021. “What Would It Take to Die Well? A Systematic Review of Systematic Reviews on the Conditions for a Good Death.” Lancet Healthy Longevity 2, no. 9: e593–e600. 10.1016/S2666-7568(21)00097-0. 36098155

