# Supplementary material for: Hospitalization and Death Acceptance: A Meta‐Ethnography of Perceptions of Hospital as a Place of Death for Nursing Home Residents
Source: Int J Older People Nurs. 2026 Oct 4;21(6):e70101. doi: 10.1111/opn.70101 (PMC13635599; doi:10.1111/opn.70101)
Supplement: Supplementary file 1 [file OPN-21-e70101-s001.docx]

**Supplemental material table 1**

*eMERGe reporting statement*

| No. | Criteria Headings | | Reporting Criteria | | Response | |
| --- | --- | --- | --- | --- | --- | --- |
| Phase 1—Selecting meta-ethnography and getting started | | | | | |  |
| *Introduction* | | | | | |  |
| 1 | | Rationale and context for the meta-ethnography | | Describe the gap in research or knowledge to be filled by the meta-ethnography, and the wider context of the meta-ethnography | | Introduction |
| 2 | | Aim(s) of the meta-ethnography | | Describe the meta-ethnography aim(s) | | Page 4-5 |
| 3 | | Focus of the meta-ethnography | | Describe the meta-ethnography review question(s) (or objectives) | | Page 4-5 |
| 4 | | Rationale for using meta-ethnography | | Explain why meta-ethnography was considered the most appropriate qualitative synthesis methodology | | Page 4-5 |
| Phase 2—Deciding what is relevant | | | | | |  |
| *Methods* | | | | | |  |
| 5 | | Search strategy | | Describe the rationale for the literature search strategy | | Page 6 |
| 6 | | Search processes | | Describe how the literature searching was carried out and by whom | | Page 6; supplementary  material table 2 |
| 7 | | Selecting primary studies | | Describe the process of study screening and selection, and who was involved | | Page 7 |
| *Findings* | | | | | |  |
| 8 | | Outcome of study selection | | Describe the results of study searches and screening | | Pages 9-11, Figure 1 |
| Phase 3—Reading included studies | | | | | |  |
| *Methods* | | | | | |  |
| 9 | | Reading and data extraction approach | | Describe the reading and data extraction method and processes | | Page 7-8 |
| *Findings* | | | | | |  |
| 10 | | Presenting characteristics of included studies | | Describe characteristics of the included studies | | Page 9-10, Table 1, Table 2 |
| Phase 4—Determining how studies are related | | | | | |  |
| *Methods* | | | | | |  |
| 11 | | Process for determining how studies are related | | Describe the methods and processes for determining how the included studies are related:  - Which aspects of studies were compared  AND  - How the studies were compared | | Pages 5,8,9; table 3 |
| *Findings* | | | | | |  |
| 12 | | Outcome of relating studies | | Describe how studies relate to each other | | Findings, Figure 2, table 3 |
| Phase 5—Translating studies into one another | | | | | |  |
| *Methods* | | | | | |  |
| 13 | | Process of translating studies | | Describe the methods of translation**:**  - Describe steps taken to preserve the context and meaning of the relationships between concepts within and across studies- Describe how the reciprocal and refutational translations were conducted- Describe how potential alternative interpretations or explanations were considered in the translations | | Page 7,8,9 |
| *Findings* | | | | | |  |
| 14 | | Outcome of translation | | Describe the interpretive findings of the translation. | | Table 3, Figure 2 |
| Phase 6—Synthesizing translations | | | | | |  |
| *Methods* | | | | | |  |
| 15 | | Synthesis process | | Describe the methods used to develop overarching concepts (“synthesised translations”)Describe how potential alternative interpretations or explanations were considered in the synthesis | | Page 8,9, Figure 2 |
| *Findings* | | | | | |  |
| 16 | | Outcome of synthesis process | | Describe the new theory, conceptual framework, model, configuration, or interpretation of data developed from the synthesis | | Findings, Figure 2 |
| Phase 7—Expressing the synthesis | | | | | |  |
| *Discussion* | | | | | |  |
| 17 | | Summary of findings | | Summarize the main interpretive findings of the translation and synthesis and compare them to existing literature | | Page 22-25 |
| 18 | | Strengths, limitations, and reflexivity | | Reflect on and describe the strengths and limitations of the synthesis:  - Methodological aspects—for example, describe how the synthesis findings were influenced by the nature of the included studies and how the meta-ethnography was conducted.- Reflexivity—for example, the impact of the research team on the synthesis findings | | Page 28 |
| 19 | | Recommendations and conclusions | | Describe the implications of the synthesis | | Page 26-29 |

**Supplementary material Table 2**

*Search strategy for MEDLINE, CINAHL, PsycINFO, and Scopus*

*Limit:*  January 2012 to March 2024

|  | **Nursing home** |  | **Mortality** |  | **Hospital** |
| --- | --- | --- | --- | --- | --- |
| **Search terms**  TI/AB | “nursing home*” OR “long term care” OR “skilled nursing facilit*” OR “residential age* care” OR RAC OR “residential age* care facilit*” OR RACF OR “housing for the elderly” OR “homes for the aged” OR “geriatric nurs*” OR “geriatric care” | **AND** | mortality OR death OR dying OR die* OR "terminal care" OR "palliative care" OR "end of life*" OR EOL OR EOLC OR "with* treatment*" | **AND** | “emergency medical servic*” OR “emergency treatment*” OR “emergency service*” OR “emergency nurs*” OR “emergency department*” OR “emergency medicine” OR “trauma servic*” OR “trauma cent*” OR “accident emergency” OR ambulanc* OR paramedi* OR “emergency medical technician” OR EMT OR prehospital OR “prehospital care” OR hospital* OR transfer OR admission |
| **Medline** MeSH | (MH "Nursing Homes+") OR (MH "Residential Facilities+") OR (MH "Skilled Nursing Facilities") OR (MH "Long-Term Care") OR (MH "Housing for the Elderly") OR (MH "Homes for the Aged") OR (MH "Geriatric Nursing") | **AND** | (MH "Mortality+") OR (MH "Hospital Mortality") OR (MH "Death+") OR (MH "Terminal Care+") OR (MH "Palliative Care") OR (MH "Hospice and Palliative Care Nursing") OR  (MH "Withholding Treatment+") | **AND** | (MH "Emergency Medical Services+") OR (MH "Emergency Service, Hospital+") OR (MH "Emergency Medical Technicians") OR (MH "Emergency Treatment+") OR (MH "Emergency Nursing") OR (MH "Emergency Medicine+") OR (MH "Emergencies") OR (MH "Trauma Centers") OR (MH "Ambulances+") OR (MH "Hospitals+") OR (MH "Patient Transfer") OR (MH "Patient Admission") |
| **CINAHL** | (MH "Nursing Homes+") OR (MH "Residential Facilities+") OR (MH "Long Term Care") OR (MH "Nursing Home Patients") OR (MH "Skilled Nursing Facilities") OR (MH "Housing for the Elderly") OR (MH "Gerontologic Nursing+") OR (MH "Gerontologic Care") OR (MH "Age Specific Care") | **AND** | (MH "Mortality+") OR (MH "Hospital Mortality") OR (MH "Death+") OR (MH "Cause of Death") OR (MH "Terminally Ill Patients+") OR (MH "Terminal Care+") OR (MH "Palliative Care") OR (MH "Hospice and Palliative Nursing") OR (MH "Euthanasia, Passive") | **AND** | (MH "Emergency Medical Services+") OR (MH "Emergency Service+") OR (MH "Emergency Treatment+") OR (MH "Physicians, Emergency") OR (MH "Emergency Nurse Practitioners") OR (MH "Emergency Nursing+") OR (MH "Trauma Centers") OR (MH "Ambulances") OR (MH "Emergency Medical Technicians") OR (MH "Prehospital Care") OR (MH "Hospitals+") OR (MH "Transfer, Discharge") OR (MH "Patient Admission") OR (MH "Emergency Patients") |
| **PsycInfo** | DE "Long Term Care" OR DE "Nursing Home Residents" OR DE "Nursing Homes" OR DE "Residential Care Institutions" | **AND** | DE "Death and Dying" OR DE "Assisted Suicide" OR DE "Euthanasia" OR DE "Mortality Rate" OR DE "Mortality Risk" OR DE "Parental Death" OR DE "Partner Death" OR DE "Sudden Death" OR DE "Terminally Ill Patients" OR DE "Treatment Withholding" OR DE "Palliative Care" | **AND** | DE "Emergency Services" OR DE "Crisis Intervention Services" OR DE "Emergency Personnel" OR DE "Fire Fighters" OR DE "First Responders" OR DE "Paramedics" OR DE "Police Personnel" OR DE "Rescue Workers" OR DE "Emergency Medicine") OR (DE "Paramedics")) OR (DE "Emergency Medicine")) OR (DE "Hospital Admission" OR DE "Psychiatric Hospital Admission")) OR (DE "Client Transfer" DE "Hospitals") |
| **Scopus**  No subject headings | Keywords as above (TI/AB) |  |  |  |  |
